# Myelin organoids for the study of Alzheimer's disease

**DOI:** 10.3389/fnins.2023.1283742

**Published:** 2023-10-24

**Authors:** Jonas Cerneckis, Yanhong Shi

**Affiliations:** ^1^Department of Neurodegenerative Diseases, Beckman Research Institute of City of Hope, Duarte, CA, United States; ^2^Irell and Manella Graduate School of Biological Sciences, Beckman Research Institute of City of Hope, Duarte, CA, United States

**Keywords:** Alzheimer's disease, myelin, myelinoids, oligodendrocytes, microglia

## Introduction

Alzheimer's disease (AD) remains a leading cause of dementia and poses a heavy burden on aging populations worldwide (Long and Holtzman, 2019). Amyloid-targeting antibodies have recently gained accelerated (aducanumab) and full approval (lecanemab) by the Food and Drug Administration, ushering in a new era of AD therapeutics targeting neuronal pathology (Shi et al., 2022). Dysfunction of glial cells has also emerged as a major factor in AD progression, contributing to neuroinflammation, impaired lipid metabolism, myelin breakdown, and other disease-associated phenotypes (De Strooper and Karran, 2016; Long and Holtzman, 2019). Given the species-specific differences between rodent models and humans, the study of glial cell dysregulation in AD has been greatly facilitated by the induced pluripotent stem cell (iPSC) technology that enables differentiation of human glial cells *in vitro* (Shi et al., 2017; Li and Shi, 2020; Chen et al., 2021b). Moreover, brain organoids—three-dimensional (3D) iPSC-derived human brain-like tissues—recapitulate intricate cell-cell interactions of the human brain that deteriorate in AD (Di Lullo and Kriegstein, 2017; Cerneckis et al., 2023). Although astrocyte- and microglia-containing brain organoids have been widely applied to study glial cell type-specific responses to AD pathology, oligodendrocyte-containing myelin organoids (myelinoids) are yet to be used to model myelin breakdown in AD (Cerneckis et al., 2023). In this opinion, we discuss recent findings on the importance of myelin dysfunction in AD pathogenesis and highlight potential applications of myelinoids to test specific hypotheses *in vitro*.

## Myelin dysfunction in Alzheimer's disease

The myelin membrane is produced by oligodendrocytes and ensheaths axons to provide electrical insulation, facilitate saltatory conduction of action potentials, and support neuronal metabolism (Lee et al., 2012; Stadelmann et al., 2019). Extensive myelination and enlargement of prefrontal white matter distinguish the human brain from other species but may also confer unique vulnerability to neurological disorders associated with myelin dysfunction (Schoenemann et al., 2005; Bartzokis, 2011, 2012). Myelin volume decreases in aging, which may contribute to the onset and progression of age-related neurodegeneration (Braak and Braak, 1996; Bartzokis, 2011). Indeed, magnetic resonance imaging (MRI) has revealed white matter deficits and inflammation in asymptomatic individuals who later progress to AD (Brickman et al., 2015; Caballero et al., 2018; Wang et al., 2019) and an association between white matter damage and classical amyloid-β (Aβ) and phosphorylated tau (p-tau) pathology (Dean et al., 2017; Wang et al., 2019). Myelin loss (Chen J. F. et al., 2021) and changes in oligodendrocyte gene expression programs (Kenigsbuch et al., 2022; Sadick et al., 2022; Murdock and Tsai, 2023) are also evident in postmortem brain tissue of AD patients and AD mouse models. Single-cell profiling of oligodendrocyte gene expression has revealed aberrant expression of genes required for myelin synthesis, impaired lipid metabolism, and a population of disease-associated oligodendrocytes (DOLs) in AD (Kenigsbuch et al., 2022; Sadick et al., 2022; Murdock and Tsai, 2023). Elucidating whether myelin dysfunction can trigger Aβ and p-tau pathology as an upstream event in AD pathogenesis would provide strong rationale for therapeutic development targeting myelin pathology at early stages of the disease (Bartzokis, 2011). Although the idea of myelin breakdown as an event upstream of Aβ and p-tau pathology was proposed by a neuroscientist George Bartzokis (1956–2014) two decades ago (Bartzokis, 2004, 2011), only recently has experimental evidence been acquired to support this hypothesis (Depp et al., 2023). Depp et al. (2023) have demonstrated that myelin-defective neurons form axonal swellings that accumulate the amyloid precursor protein (APP) processing machinery, leading to increased production of Aβ. These findings suggest that breakdown of myelin can act as an upstream cause of classical AD pathology, whereas limiting myelin dysfunction might be a promising therapeutic strategy to treat cognitive decline and AD (Depp et al., 2023). Indeed, pharmacologically enhancing remyelination with clemastine improves cognitive functions of aging mice (Wang et al., 2020).

Genetic risk factors for AD identified in genome-wide association studies (GWAS) (Wightman et al., 2021), such as the E4 variant of apolipoprotein E (*APOE4*), also contribute to myelin dysfunction. The detrimental effects of *APOE4*, the strongest genetic risk factor for AD, have been characterized in different brain cell types, including oligodendrocytes (Lin et al., 2018; Blanchard et al., 2022; Martens et al., 2022). In *APOE4* carriers, dysregulation of lipid metabolism and cholesterol accumulation in oligodendrocytes lead to impaired myelination, including formation of thinner myelin sheaths, as compared to *APOE3* carriers (Blanchard et al., 2022). Interestingly, 2-hydroxypropyl-β-cyclodextrin, a compound that promotes cholesterol transport, ameliorates cholesterol accumulation in iPSC-derived *APOE4* oligodendrocytes, promotes myelination *in vitro* and *in vivo*, and improves cognition of *APOE4* targeted replacement mice (Blanchard et al., 2022). Conditional removal of *APOE4* in neurons is associated with decreased expression of DOL cluster genes and improved myelination, indicating that ApoE4 secreted by other cell types also influences oligodendrocyte phenotypes (Koutsodendris et al., 2023). We have recently shown that the clusterin (*CLU*, also known as apolipoprotein J, *APOJ*) isoform encoded by the “C” allele of the rs11136000 polymorphism impairs oligodendrocyte progenitor cell (OPC) proliferation and myelination through paracrine signaling from astrocytes (Liu et al., 2023). In particular, iPSC-derived astrocytes carrying the “C” allele exhibit increased activity of the interferon response pathway and higher secretion of the C-X-C motif ligand 10 (CXCL10) as compared to astrocytes carrying the “T” allele (Liu et al., 2023). These findings highlight the involvement of paracrine signaling and cell-cell interactions in myelin breakdown that can be investigated further using myelinoids.

As professional phagocytes, microglia play a major role in myelin homeostasis and clearance of myelin debris (Safaiyan et al., 2016; McNamara et al., 2023). A unique profile of white matter-associated microglia (WAM), including their increased phagocytic activity and altered lipid metabolism, suggests that the myelin-rich environment shapes microglial identity and gene expression (Safaiyan et al., 2021). A partial overlap between gene expression profiles of WAM and disease-associated microglia (DAM), such as upregulation of *APOE* expression, may indicate a common response to disease-associated debris (Safaiyan et al., 2021). Although clearance of myelin debris and recycling of lipid species, such as cholesterol, promote myelin repair, excessive myelin debris overwhelms microglia (Safaiyan et al., 2016; Cantuti-Castelvetri et al., 2018; Depp et al., 2023). Accumulation of high-molecular weight myelin fragments in microglia leads to formation of insoluble lipofuscin, which likely further burdens the lysosomal compartment required for efficient breakdown of AD-associated debris (Depp et al., 2023). Interestingly, microglia engage myelin debris at the expense of amyloid plaque clearance, which may worsen classical AD pathology (Depp et al., 2023).

Overall, defining how Aβ and p-tau pathology, genetic and other risk factors for AD, and deterioration of cell-cell interactions interplay to drive myelin dysfunction may reveal novel targets for therapeutic development for AD. In the remainder of this opinion, we discuss the potential applications of myelinoids to study these phenotypes.

## Myelinoids as a model of myelin biology

Human iPSCs can be differentiated into oligodendrocytes by mimicking the *in vivo* developmental trajectory of oligodendroglia (Douvaras and Fossati, 2015; Li et al., 2018; Liu et al., 2023). Similarly, exposure of differentiating brain organoids to factors that promote oligodendrocyte fate, including thyroid hormone (T3), insulin-like growth factor 1 (IGF-1), and platelet-derived growth factor AA (PDGF-AA), yields oligodendrocyte-containing myelinoids (Madhavan et al., 2018; Kim et al., 2019; Marton et al., 2019; James et al., 2021; Feng et al., 2023). Accelerated differentiation of oligodendrocytes in neural spheroids can also be achieved by overexpression of master transcription factors involved in oligodendrogenesis, *SOX10* and *OLIG2* (Ma et al., 2022). Several groups have developed cortical myelinoids patterned toward the forebrain identity by dual SMAD inhibition (Madhavan et al., 2018; Kim et al., 2019; Marton et al., 2019); these organoids contain OPCs and myelinating oligodendrocytes that ensheath axons in the 3D space. Furthermore, James et al. (2021) have shown that myelinoids patterned toward ventral spinal cord identity, where oligodendrogenesis begins during development, exhibit advanced myelination patterns, including myelin internodes, paranodal junctions, and nodes of Ranvier. Although cortical myelinoids might be more suitable for modeling AD, improved myelination of spinal cord myelinoids makes them an attractive alternative to investigate the specificities of myelin sheath maintenance and dysfunction. An important advantage of modeling AD with iPSC-derived myelinoids is their human origin that enables the study of human-specific phenotypes that may not be recapitulated in animal models (Fang et al., 2022; Gargareta et al., 2022). Moreover, varying observations of myelin dysfunction in AD mouse models across different studies (Zhang et al., 2020; Chen J. F. et al., 2021; Depp et al., 2023) further support the use of myelinoids to clarify disease-relevant phenotypes. Feng et al. (2023), James et al. (2021), and Madhavan et al. (2018) have shown that myelinoids recapitulate key aspects of genetic myelin disorders, including Canavan disease (CD) and Pelizaeus–Merzbacher disease. For example, exposure of aspartoacylase (ASPA)-deficient myelinoids derived from iPSCs of CD patients to N-acetyl-aspartate (NAA) induces extensive damage to myelin sheaths, mimicking spongy degeneration of the white matter in CD patients (Feng et al., 2023). Furthermore, myelinoids are responsive to pharmacological intervention; for example, pro-myelinating drugs ketoconazole and clemastine promote oligodendrogenesis in myelinoids (Madhavan et al., 2018). These findings indicate that myelinoids can be applied to study myelin dysfunction in human diseases, including AD, and develop novel therapeutics for modulating myelination.

## Modeling Alzheimer's disease with myelinoids

**Figure 1:**
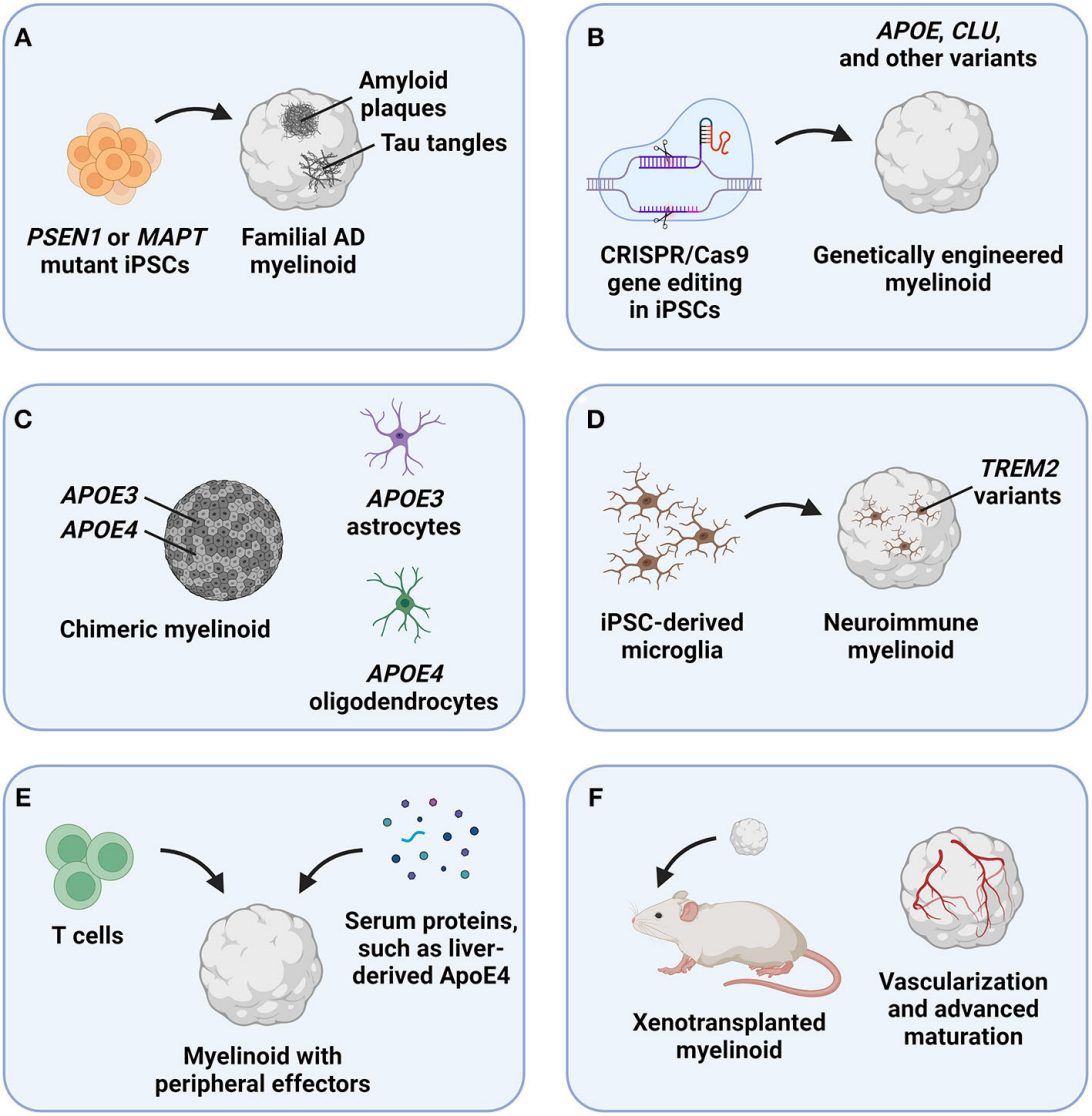
Potential applications of myelinoids to study myelin dysfunction in Alzheimer's disease (AD). **(A)** The effects of classical AD pathology, including amyloid plaques and tau tangles, on oligodendroglia and myelin health can be studied by deriving myelinoids from induced pluripotent stem cells (iPSCs) carrying disease-causing mutations, such as those in the presenilin 1 (*PSEN1*) and microtubule-associated protein tau (*MAPT*) genes. **(B)** The effects of genetic risk factors for late-onset AD, such as variants of the apolipoprotein E (*APOE*) and clusterin (*CLU*) genes, can be studied in myelinoids derived from CRISPR/Cas9-edited iPSCs. **(C)** To discern between cell-autonomous and non-autonomous effects of genetic risk factors expressed in multiple cell types, such as *APOE*, chimeric myelinoids with cell type-specific genotypes can be derived. **(D)** The roles of microglia in clearing myelin debris and recycling myelin lipids can be studied in neuroimmune myelinoids that contain iPSC-derived microglia. **(E)** The effects of peripheral factors, such as immune cells and serum proteins, can be modeled by exposing myelinoids to these effectors *in vitro*. **(F)** To achieve advanced myelinoid maturation, these organoids can be xenotransplanted into rodent models.

Myelinoids should be first employed to assess the effects of classical AD pathology—Aβ and p-tau aggregates—on oligodendrocyte and myelin homeostasis (Figure 1A). Aβ and p-tau pathology robustly develops in brain organoids derived from iPSCs of patients carrying disease-causing mutations, such as those in presenilin 1 (*PSEN1*) and microtubule-associated protein tau (*MAPT*) genes (Cerneckis et al., 2023). Aβ and p-tau pathology can also be induced acutely by treating brain organoids with a small-molecule compound Aftin-5 (Pavoni et al., 2018) and by delivering a mutant *MAPT* expression cassette using adeno-associated virus (Shimada et al., 2022), respectively. Whether acute demyelination can also induce Aβ and p-tau formation can be assessed by treating myelinoids with demyelinating compounds, such as lysolecithin (Marton et al., 2019). These models of myelin dysfunction may clarify the interplay between classical AD pathology and myelination. It will also be important to characterize the OPC pool, such as their proliferation and differentiation capacity in the presence of toxic Aβ and p-tau aggregates, and define oligodendrocyte gene expression profiles that may reveal distinct cellular states upon Aβ and p-tau challenge in myelinoids. The impact of myelin breakdown on neuronal network connectivity in myelinoids can be assessed using multi-electrode array (MEA) analysis with ever improving spatiotemporal resolution of MEA devices (Floch et al., 2022; Huang et al., 2022a).

The roles of genetic risk factors for AD in myelin homeostasis can be defined by deriving genetically engineered myelinoids that express genetic risk variants, such as *APOE4* (Figure 1B). As a transporter of cholesterol, ApoE plays an important role in the recycling of myelin lipids and in myelin repair; notably, neurons and astrocytes exhibit dysregulated cholesterol biosynthesis and accumulate cholesterol in *APOE4* brain organoids (Zhao et al., 2023). Therefore, myelinoids with different ApoE isoforms can be used to characterize how each isoform affects lipid metabolism and myelination that require coordinated interactions and metabolite exchange between different brain cell types. Because *APOE* is expressed in multiple brain cell types, cell autonomous and non-autonomous effects of ApoE could be assessed using chimeric myelinoids, where only a specific cell type expresses *APOE4* (Figure 1C) (Huang et al., 2022b). Another effector involved in lipid metabolism and implicated in AD pathogenesis, triggering receptor expressed on myeloid cells 2 (*TREM2*), is expressed in microglia and mediates extracellular lipid sensing (Deczkowska et al., 2020). However, the lipid sensing ability of the R47H isoform of TREM2 is impaired (Wang et al., 2015). To study the effects of *TREM2* and other genetic risk variants specific to microglia, neuroimmune myelinoids can be generated by seeding iPSC-derived microglia onto the myelinoid to allow microglia infiltration and interaction with other cell types in the organoid (Figure 1D) (Cerneckis and Shi, 2023a; Cerneckis et al., 2023). Given the major roles that microglia play in clearing myelin debris, recycling lipids, and mediating neuroinflammation in AD, neuroimmune myelinoids will serve as a robust platform to define the interplay between microglia and myelin dysfunction in AD. It will also be important to clarify whether disruption of lipid and myelin homeostasis can drive neuroinflammation by activating microglia or astrocytes and which lipid species released with myelin debris are inflammatory. For example, astrocyte activation is evident in myelin-rich regions in a mouse model of sulfatide deficiency, contributing to neuroinflammation (Qiu et al., 2021).

Peripheral effectors, such as immune cells and serum proteins have been recently implicated in AD pathogenesis as well (Gate et al., 2020; Chen et al., 2021a; Kaya et al., 2022; Liu et al., 2022). For example, liver-derived ApoE4 impairs cognition in a mouse model (Liu et al., 2022), whereas exposure of brain organoids to human serum precipitates Aβ and p-tau pathology (Chen et al., 2021a). Moreover, oligodendrocytes co-localize with CD8^+^ T cells in the aging mouse brain, leading to oligodendrocyte activation and death (Kaya et al., 2022). To gain a deeper understanding on how peripheral effectors impact myelin homeostasis in AD, myelinoids can be co-cultured with primary or iPSC-derived immune cells (Sommer et al., 2018), exposed to other peripheral factors (Chen et al., 2021a), or assembled into state-of-the-art microfluidic organoids-on-a-chip with a separated peripheral compartment (Figure 1E) (Cho et al., 2021; Ronaldson-Bouchard et al., 2022).

Finally, various myelinoids discussed earlier can be transplanted *in vivo* to promote advanced myelinoid maturation and vascularization (Figure 1F). Indeed, brain organoid transplantation paradigm has recently emerged as a powerful approach to achieve improved organoid maturation, revealing subtle disease phenotypes that are not recapitulated *in vitro* (Mansour et al., 2018; Revah et al., 2022; Cerneckis and Shi, 2023b; Schafer et al., 2023). It can be expected that xenotransplanted myelinoids will contain a robust pool of OPCs and mature oligodendrocytes as well as exhibit improved myelination patterns, making them especially suitable for modeling myelin dysfunction in AD. Transplantation of myelinoids carrying causal mutations or genetic risk factors for AD into wild-type mice can be performed to improve myelinoid maturation and study organoid-specific phenotypes. Alternatively, myelinoid transplantation into transgenic AD mouse models will enable the study of complex disease phenotypes, such as Aβ propagation into the myelinoid, dysfunction of organoid-invading murine vasculature, and the effects of blood-derived factors or systemic perturbations on AD progression.

## Limitations of modeling Alzheimer's disease with myelinoids

It is also important to consider various limitations of the brain organoid technology for modeling AD, so that appropriate experimental controls can be utilized (Cerneckis et al., 2023). Most notably, iPSC-derived cellular models, including brain organoids, exhibit fetal-like gene expression signatures and represent early human brain development, whereas AD is an age-related neurodegenerative disease (Cornacchia and Studer, 2017). Furthermore, brain organoids are a simplified model of the human brain and often lack important cellular components, such as endothelial cells, that can also contribute to AD progression (Cerneckis et al., 2023). Therefore, myelinoids that lack aging-associated phenotypes and complete cellular diversity may not recapitulate all disease-relevant phenotypes. Nonetheless, the effects of causal disease mutations, genetic and non-genetic risk factors, and specific cell types on AD progression have been successfully modeled using brain organoids (Cerneckis et al., 2023). Limited yet sufficient complexity of brain organoids may even be advantageous to pinpoint key dysregulated pathways that drive AD pathogenesis. If necessary, neurodegenerative phenotypes can be amplified by exposing myelinoids to a combination of genetic and non-genetic risk factors for AD, whereas additional cell types can be introduced by established techniques (Di Lullo and Kriegstein, 2017). Importantly, novel phenotypes obtained from myelinoid studies should be validated by a comparative analysis of primary human brain tissue whenever possible.

## Discussion

Although AD has historically been considered a gray matter disease, emerging evidence indicates concurrent white matter dysfunction during AD progression. The versatile myelinoid platform offers unprecedented access to human brain myelination models that can be tailored to address specific hypotheses pertaining to myelin dysfunction in AD. For example, microglia can be incorporated into myelinoids described in Figures 1A–C, E, F to study the interplay between microglia, various genetic and non-genetic risk factors that contribute to AD progression, and myelin dysfunction. Myelinoids can also be combined with other technological advancements, such as high-throughput drug screening and high-content imaging assays, to systematically assess disease-relevant pathology and drug efficacy (James et al., 2021; Park et al., 2021). Overall, we anticipate that myelinoids will facilitate the elucidation of human-specific molecular mechanisms and cellular phenotypes governing oligodendrocyte function and advance the development of novel therapeutics targeting impaired myelination in AD.

## Author contributions

JC: Writing—original draft, Writing—review and editing. YS: Writing—review and editing.
